# General practitioner workforce planning: assessment of four policy directions

**DOI:** 10.1186/1472-6963-10-148

**Published:** 2010-06-02

**Authors:** Conor Teljeur, Stephen Thomas, Fergus D O'Kelly, Tom O'Dowd

**Affiliations:** 1 Department of Public Health and Primary Care, Trinity College Centre for Health Sciences, AMiNCH, Tallaght, Dublin 24, Ireland; 2 Health Policy & Management, Room 0.21, 3-4 Foster Place, College Green, Dublin 2, Ireland

## Abstract

**Background:**

Estimating the supply of GPs into the future is important in forecasting shortages. The lengthy training process for medicine means that adjusting supply to meet demand in a timely fashion is problematic. This study uses Ireland as a case study to determine the future demand and supply of GPs and to assess the potential impact of several possible interventions to address future shortages.

**Methods:**

Demand was estimated by applying GP visit rates by age and sex to national population projections. Supply was modelled using a range of parameters derived from two national surveys of GPs. A stochastic modelling approach was adopted to determine the probable future supply of GPs. Four policy interventions were tested: increasing vocational training places; recruiting GPs from abroad; incentivising later retirement; increasing nurse substitution to enable practice nurses to deliver more services.

**Results:**

Relative to most other European countries, Ireland has few GPs per capita. Ireland has an ageing population and demand is estimated to increase by 19% by 2021. Without intervention, the supply of GPs will be 5.7% less than required in 2021. Increasing training places will enable supply to meet demand but only after 2019. Recruiting GPs from overseas will enable supply to meet demand continuously if the number recruited is approximately 0.8 per cent of the current workforce per annum. Later retirement has only a short-term impact. Nurse substitution can enable supply to meet demand but only if large numbers of practice nurses are recruited and allowed to deliver a wide range of GP services.

**Conclusions:**

A significant shortfall in GP supply is predicted for Ireland unless recruitment is increased. The shortfall will have numerous knock-on effects including price increases, longer waiting lists and an increased burden on hospitals. Increasing training places will not provide an adequate response to future shortages. Foreign recruitment has ethical considerations but may provide a rapid and effective response. Increased nurse substitution appears to offer the best long-term prospects of addressing GP shortages and presents the opportunity to reshape general practice to meet the demands of the future.

## Background

Internationally, general practice workforce modelling has only been partly successful with governments struggling to accurately estimate future needs [1,2]. Due to the lengthy training process for medicine, adjusting supply to meet demand in a timely fashion poses difficulties. General practice is undergoing structural and demographic changes which add further complexity to estimating future supply. Compared to many clinical specialities, general practice offers greater flexibility in working arrangements which has made it more attractive to female clinicians [3,4] with many countries seeing an increased number of females in the specialty [5]. There are concerns that female GPs tend to work shorter hours and that feminisation equates to reduced service provision. The increasing feminisation of general practice has been well documented but the ageing of the workforce is often overlooked. A marked ageing of the GP workforce has been noted in Australia [6] and the Netherlands [7]. As the older GPs retire, they will be replaced by a younger cohort that favour shorter hours and earlier retirement. Changes in the workforce profile will undoubtedly have implications for availability of GP services. When considered in the context of ageing populations in many Western nations, there will be a need for increased numbers of GPs and many countries may be faced with workforce shortages.

There have been substantial changes in the age, gender and work patterns of GPs in Ireland over the last 25 years [8]. Graduates increasingly favour flexible hours and earlier retirement [9]. Concern has been expressed that if trends continue there will be serious problems with the availability of GP services over the next decade. The existing workforce is 44% female compared to only 15% in the early 1990s. The mean age of GPs is 47.8 years. Fifteen percent of GPs are over 60 although the vast majority of older GPs are male. The percentage GPs in rural practice has declined from 33% in the early 1990s to less than 22% in 2005 [10]. Much of the decrease in rural practice has translated into an increase in GPs in a mixed urban/rural setting. Rural practice accounts for more than a third of GPs over 60. More than 90% of GPs have a contract with the state to provide care to some or all of their patients. The state contracts stipulate a retirement age of 70 although few GPs continue past the age of 65. The vast majority of new GPs have undergone vocational training in Ireland with some having been trained in the UK. Vocational training places in Ireland are increasing from 120 to 160 in 2010. Eighty four percent of GPs under 50 years of age have had formal GP training compared to only 32% of GPs aged 50 years and over. There is no formal recruitment of GPs from abroad.

The Irish general practice workforce is small and experiences little inward migration of workers from abroad. As such the main factors influencing supply can more easily be determined than in a larger country, particularly over a relatively short horizon. The aim of this study was to use Ireland as a case study to generate models for policy interventions to address future GP workforce shortages.

## Methods

### Setting

Ireland has a dispersed population of 4.2 million and just over 2,500 self-employed GPs who hold State contracts to provide means tested medical care to those entitled to such care. The private provision of health care services plays an important role in the Irish health system. This means that most patients pay their GPs directly for their services, on a fee-for-service basis, which is unusual for Western Europe; this works out at €50 per consultation. A minority of patients, those on low incomes and the majority of those aged 70 and over, are entitled to "medical cards" which allow free access to GPs and hospitals [11]. This covers around 30% of the population. The government contracts GPs on a capitation basis to treat medical card holders. GPs with a contract to treat "medical card" patients must have vocational training. The number of vocational training places is capped with over 300 applying for 160 places annually across 13 accredited vocational training courses.

### Demand

Annual frequency of visits to GP by age and sex was estimated using survey data collected nationally in 2002 [11]. Mean visit rates were computed for a number of age categories to be applied to population figures. Demand was projected based on age-sex rates of GP attendance applied to population projections. The population projections for the period 2009 to 2021 were obtained from the Central Statistics Office (CSO) [12]. The projections were available for two distinct international migration assumptions, M0 and M2, which predict zero and moderate growth, respectively. Due to the recent economic downturn it is unlikely that there will be substantial inward migration and the assumption of zero growth due to migration has been used in our calculations.

### Supply

With the exception of mortality, all parameters were derived from data gathered in two national surveys [8,9]. The Structure of General Practice in Ireland report surveyed a nationally representative sample of 476 GPs (response rate 87%) on a wide range of topics including demography, work practice, services provided, practice structure and education [8]. The census of vocational training graduates surveyed 245 graduates from the years 1997 to 2003 (response rate 75%) on work practice, career development, family demands and also reasons for leaving general practice where applicable [9]. The two national surveys provide a comprehensive overview of working behaviour in Irish GPs. Information on mortality was obtained from national vital statistics data using the mortality rate for higher professionals, a category that includes medical practitioners [13]. It is assumed that holidays and maternity leave are fully covered by locums. The base unit of GP service provision is a session which represents a half day in practice. The number of patients seen over the course of a session varies according to doctor, practice, local demography and case complexity.

### Intervention models

Attempts to increase the supply can be achieved by either boosting entrants to the workforce, increasing productivity or by reducing the numbers exiting the workforce. This study has concentrated on interventions to improve recruitment and also the potential for deferring retirement. It is assumed that GP productivity is already high and that further increases could have significant cost implications and may have a detrimental impact on the quality of the service provided. As such, an intervention regarding increased productivity has not been considered. This study considered four main policy interventions to address future GP workforce shortages using the following assumptions:

1. Increasing the number of GP vocational training places by 20% in 2011. It was assumed that all training places will be filled and that the age-sex distribution of applicants will remain unchanged over the study period. Training places were increased by 25% in 2010 and will put pressure on capacity at the training courses. A further increase of 20% in 2011 is considered feasible.

2. Recruiting GPs from abroad. It was assumed that GPs recruited from abroad would be 90% male and typically 5 years older than graduates from the Irish training schemes. It is assumed that male GPs are more likely to migrate and that they will not do it immediately after graduation in their home country. Two alternatives were tested: recruitment of 10 and 20 GPs per annum from outside Ireland, respectively. This represents an approximate doubling of the numbers of GPs entering Ireland annually. Given the lack of track record in foreign recruitment this represents a reasonable aspiration.

3. Incentivising later retirement for existing GPs. Late retirement was applied to all GPs that indicated a projected retirement age of 65 or younger. Two scenarios were tested: an average of an additional one year (range 0-5 years based on Poisson distribution) and two years (range 0-8 years based on Poisson distribution), respectively. Older GPs are at increased risk of burn-out and may not be easily encouraged to continue in practice hence the choice of conservative extensions to working life.

4. Increasing nurse substitution to enable practice nurses to deliver more services. It was assumed that recruited nurses would be of a similar age structure to GP graduates but that they would be 90% female. It was assumed that a practice nurse would not be equivalent to a full time equivalent (FTE) GP. Two scenarios were tested in which nurses were equivalent to 0.25 and 0.5 FTE GPs, respectively. In the former scenario, a practice nurse will be able to treat a quarter of the patients that a GP of equivalent age and sex might provide. Equivalence depends on factors such as the ability to prescribe. Both scenarios are based on an annual increase of approximately 5% of the current number of practice nurses. In the absence of applicable data on equivalence two scenarios are tested to provide guidelines as to the potential impact of nurse substitution.

Each of these interventions was assessed in terms of how it would impact on the number of sessions delivered relative to the number required per demand.

### Statistical methodology

Methods used for health care worker forecasting typically fall into three broad categories: population-based models, micro or macro economic models, and operations research methods and can be further sub-categorised into supply side and demand side approaches [14]. In modelling based on either supply or demand there is an implicit assumption that demand will adjust to meet supply or vice versa. Simple manpower to population ratios are popular in supply side modelling but can overlook important demographic factors such as an ageing population or part-time workers.

A number of studies have projected the supply of clinicians based on economic activity on the grounds that historically there is strong correlation between the two [15,16]. However, using economic activity replaces one forecasting problem with another as future economic activity would need to be estimated and adds an additional degree of uncertainty. Due to the current number of GP training places in Ireland and the high demand for those places it is a reasonable assumption that the number of graduates will be equal to the number of available places.

Supply is modelled as a function of vocational training graduates, retention, sessions worked, retirements and deaths. With the exception of mortality, all parameters are allowed to vary according to random distributions derived from the survey data (see Table 1 for the list of parameters with means and standard deviations). In a single simulation a number of steps are applied to each year from 2009 to 2021: GPs who reached retirement age or died are removed from the database; age and sex distributions are applied to graduates and a retention rate applied; graduates are given a retirement age sampled from the survey data for individuals of the same sex and no more than years older or younger; all GPs are given a number of sessions sampled from the survey data for individuals of the same sex and no more than 10 years older or younger. It is assumed that productivity in terms of sessions will not change significantly over the 13 year forecasting period. The calculation for a given year *t *can be summarised as follows:

Where:

workforce = the active GP workforce

graduates = the graduates of Irish vocational training courses in year *t*

migrants = entrants by routes other than Irish vocational training (e.g. UK training)

foreign = entrants on foot of specific foreign recruitment

nurse = entrants to general practice through nurse substitution

dropouts = those leaving general practice

retirement = those who have reached retirement age

mortality = those who died in year *t*

**Table 1:** Parameters included in the workforce model

Parameter	Data
Existing workforce^1^	Age-sex distribution of current workforce
Vocational training graduates^2^	Count (160 per annum)^4^, proportion male (mean 0.3 and standard deviation 0.05), age (mean 32.2 and standard deviation 2.4)
Migrants^1^	Count (15 per annum), proportion male (mean 0.75 and standard deviation 0.05), age (mean 34.9 and standard deviation 2.3)
Foreign recruits	Count^5^, proportion male (mean 0.9 and standard deviation 0.02), age (mean 36.8 and standard deviation 2.4)
Nurse substitutes	Count^6^, proportion male (mean 0.1 and standard deviation 0.02), age (mean 35.0 and standard deviation 2.4)
Sessions^1^	Sessions delivered by age and sex (overall mean 8.6 and standard deviation 2.5)
Drop-out^2^	Proportion drop-outs applied to graduate entrants (mean 0.1 and standard deviation 0.02)
Retirement^1^	Intended retirement age by age and sex (overall mean 63.2 and standard deviation 4.9)
Mortality^3^	Probability of death by age for higher professionals (ranging from 0.0006 at age 28 to 0.12 at age 80)

1. Survey of GPs [8]

2. Survey of GP training graduates [9]

3. Central Statistics Office mortality data [13]

4. Intake is 183 per annum from 2011 for intervention with increased training places.

5. There are 10 and 20 foreign recruits per annum for relevant interventions, otherwise zero.

6. There are 50 nurse substitutes per annum for relevant interventions, otherwise zero.

Note: data sampled from survey results, means and standard deviations shown for information

When calculating the number of sessions delivered by the workforce, sessions delivered by trainees were included. To estimate a stable result for each simulation model, estimations were repeated 1000 times with the median, 2.5 percentile and 97.5 percentiles recorded for each year for each of the supply variables of interest. Simulations were programmed and executed in R [17].

## Results

There are approximately 59 GPs per 100,000 persons in Ireland, similar to Portugal and Sweden but below the EU average of 100 per 100,000 persons reported by the WHO [18].

### Demand

There were approximately 13.4 million GP visits in 2009 with visit rates being highest amongst the over 60s. Population projections show an ageing population with the percentage over 60 rising from 15.7% at present to 19% in 2021. The demand for GP visits is projected to increase to 15.3 million visits per annum by 2021. The increase in demand for patient visits will be approximately 19% of current demand. When converted into GPs this equates to a demand for 3010 GPs in 2021, based on the predicted age-sex structure and productivity of GPs in 2021.

### Supply

Without additional training places, the number of GPs will remain relatively stable until 2014 after which retirements will diminish and the effects of the recent increase in training places will result in increased GP numbers. The number of GPs lost to retirement and mortality will peak between 2013 and 2016 after which the current ageing cohort will have largely retired. The projected change in GP numbers translates to an increase from 59 GPs per 100,000 at present to 59.5 by 2021. When assessed as a percentage of demand, supply in 2021 will be 5.7% less than is required.

If the intake to training schemes is increased from 160 to 183 in 2011 then the number of GPs will begin to increase from 2014 onwards as the numbers graduating exceed the numbers who retire or die. The increase in GPs will be sufficient to improve the GPs per capita to 62.9 in 2021. Under the scenario of increased training places, the 95% confidence interval for supply encompasses demand in 2021.

Recruiting qualified GPs from abroad will have an immediate impact as, unlike expanding the training schemes, there will be no lag due to training. Only by recruiting 20 GPs per annum will supply meet demand continuously through to 2021 by which point there will be approximately 230 foreign trained GPs working in Ireland through this recruitment method, representing 8% of the workforce. Recruiting 10 and 20 GPs per annum will result in supply meeting demand in 2021.

Incentivising later retirement has a modest impact on the number of GPs. Later retirement does allow supply to initially meet demand but by 2016 it falls short. An average of an additional year decreases the supply shortfall to 4.3% in 2021, compared to 5.7% if no intervention is made. An average of an additional two years further reduces the supply shortfall to 2.4% in 2021.

The impact of nurse substitution is directly dependent on what proportion of GP tasks can be provided by nurses. Of the two scenarios tested, both will enable supply to meet demand but this will require an annual increase of 5% on the current number of practice nurses.

At present 3.7% of sessions are provided by trainees. With no change in the intake to training schemes, the contribution made by trainees will increase to 5.5% by 2014 as the impact of recent training increases peak and remain stable thereafter. If training places are increased, the contribution of trainees will increase to over 6.5% by 2014 but diminish slightly thereafter as the total number of GPs increases.

**Figure 1:**
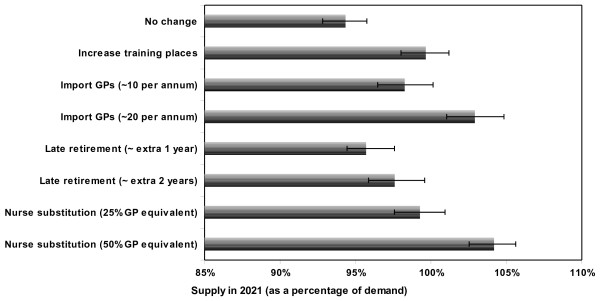
**Supply as a percentage of demand in 2021 for each intervention scenario**. Note: error bars indicate confidence bounds around supply estimates.

The impact of each of the potential interventions is given in table 2 below for three time points: 2011, 2016 and 2021. Supply as a percentage of demand in 2021 for each scenario is shown in figure 1.

**Table 2:** Impact of different interventions on the GP workforce

Intervention model			Year					
			
			2011		2016		2021	
			GPs	(% of demand)*	GPs	(% of demand)	GPs	(% of demand)
***Demand***								
	Numbers needed to meet population demand		2626	-	2825	-	3010	-

***Supply***								
	Continue current method of GP recruitment		2560	(97.6)	2629	(93.3)	2828	(94.3)
	Increase training places by 20% in 2011		2560	(97.6)	2660	(94.3)	2988	(99.7)**
	Import GPs from abroad	(average 10 per annum)	2587	(98.7)**	2698	(95.8)	2938	(98.3)**
		(average 20 per annum)	2614	(99.8)**	2781	(98.9)**	3064	(102.9)**
	Late retirement	(average extra 1 year)	2611	(99.6)**	2692	(95.4)	2880	(95.7)
		(average extra 2 years)	2652	(101.0)**	2751	(97.5)	2934	(97.6)
	Nurse substitution (average 50 nurses per annum)	(nurse equivalent to 0.25 GPs)	2704	(98.8)**	3009	(96.5)	3428	(99.3)**
		(nurse equivalent to 0.50 GPs)	2706	(100.4)**	3006	(99.9)**	3426	(104.3)**

* Supply is estimated as a number of GPs but also shown as a percentage of the GPs required as estimated by demand (shown in the first line of the table)

** Indicates that confidence bounds encompass 100% - in these cases supply could meet demand

The percentage female GPs stands at 45% in 2009 but this will increase to 62% in 2021 if the current method of GP recruitment continues. Female GPs tend to work fewer clinical sessions than their male counterparts so the change in male to female ratio will result in fewer clinical sessions per GP than provided at present. If productivity is measured by the total number of sessions delivered per GP then the reduction due to feminisation between now and 2021 is under 2% [19]. The GP demography in 2021 is given in Table 3. The mean age of GPs will reduce and the extent of the reduction is largely unaffected by the different interventions. Increased training places will result in a modest reduction whereas later retirement will cause a small increase in mean age. The absolute number of GPs over the age of 60 years will only increase if later retirement is incentivised. In all cases, older GPs will form a much smaller portion of the workforce than the current 15%. The percentage female GPs will reduce if foreign recruitment of GPs is introduced, and will increase if nurse substitution is adopted.

**Table 3:** GP demographics in 2021 under each intervention

Intervention model		Workforce demographics in 2021		
	
		Mean age	GPs over 60 (%)	Female GPs (%)
Continue current method of GP recruitment		44.2	8.3	61.9
Increase training places by 20% in 2011		43.7	7.8	62.3
Import GPs from abroad	(average 10 per annum)	44.1	8.0	59.5
	(average 20 per annum)	44.1	7.7	57.6
Late retirement	(average extra 1 year)	44.6	9.9	61.2
	(average extra 2 years)	45.0	11.6	61.0
Nurse substitution (average 50 nurses per annum)	(nurse equivalent to 0.25 GPs)	43.9	6.8	66.8
	(nurse equivalent to 0.50 GPs)	43.9	6.9	66.8

In 2007, the most recent year with comparable data, there were an estimated 12.9 million GP attendances compared to 5.3 million acute hospital attendances in Ireland [20]. A shortfall of 1% in GP supply would entail unmet demand for 129,000 GP visits. Should these visits go to hospitals instead, it would equate to a 2.3% increase in demand for hospital services. Without intervention there will be a 5.7% shortfall in the supply of GP services in 2021 which could translate to a 13.1% increase in demand for hospital services. The hospital context is already one of overcrowding with acute hospital bed occupancy at over 85% nationally.

## Discussion

If the future supply of GPs in Ireland is considered in isolation, it might appear, at least at first sight, that there will be moderate improvements. However, when demand and supply are analysed together, it becomes clear that there will be a significant deficit in the supply of GPs in the near future. This study has assessed four possible policy interventions to address the GP shortfall. Recruitment of qualified GPs from overseas and nurse substitution appear to offer the best prospects for boosting the supply of general practice services to meet demand. Increasing the number of practice nurses also has the potential to overcome shortfalls although large numbers of nurses will be required for this to be a successful alternative.

In the absence of any policy intervention it is probable that the supply of GPs will follow the predictions based on a stable trainee intake. The shortfall in supply will be noticeable and will potentially have knock-on effects including price increases, longer waiting lists and an increased burden on hospitals (primarily Emergency Departments [21]) as patients seek alternative modes of care. Higher demand in relation to supply will push up prices for GP services and this in turn may exclude those lower income families who do not have medical cards. Further with GPs closing their lists there may be access problems particularly for those patients in relatively unprofitable areas.

Although there has been substantial decline in rural general practice in Ireland, it is unclear if this trend will continue or if GPs will begin to move back into those underserved areas. Rural practices are frequently single-handed, which is less appealing to younger GPs and there may well be insufficient demand to justify multi-handed practices. The growth in practices with a mixed urban/rural client base may be indicative of rural patients increasingly being forced to travel greater distances into towns where previously there may have been a more local GP service. Whether or not an increase in the workforce might improve competition sufficiently to drive GPs to locate in such underserved areas is open to debate. It is likely that specific incentives might be required to boost supply in some rural areas. No data were available on the distribution of GPs relative to deprived areas although the provision of government contracts to treat those with low incomes provides some incentive for GPs to locate in deprived areas.

In addition to the delivery of primary health care, GPs have a gate-keeping function that controls access to hospitals and, in turn, can control health care costs. Although some patients bypass the GP and enter the hospital system directly through the Emergency Department, most patients still enter hospital via the GP. In the event of a significant shortage of GPs, increased numbers of patients may begin to enter hospitals directly, precipitating a crisis in secondary care. The longer waiting times for hospital care may rapidly translate into under-treatment in the short term and lengthier hospital stays in the longer term. With a 1% shortfall in GP supply potentially equating to a 2.4% increase in demand for hospital services, it is clearly in the interests of health service providers to ensure an adequate supply of GPs.

The 2001 'Primary Care Strategy' promoted a reform and enhancement of primary care that would result in an increased need for general practitioners [22]. Partly in response to the 'Primary Care Strategy', the ageing population and the changing work patterns of GPs, the 'Buttimer report' in 2006 recommended that training places be increased from 120 to 150 per annum [23]. Training places have recently been expanded with an intake of 160 in 2010. A further increase of 20% will be sufficient for supply to meet demand in 2021 although there will be an undersupply for many of the preceding years. It is questionable if a second expansion of places can be achieved so soon after the recent increase. Large increases in funding for medical education are not unprecedented internationally. Over six years the intake into UK medical schools increased by 60% [24]. Nevertheless, in the current economic climate it seems unlikely that funding will become available to further expand the existing vocational training schemes, much less develop new schemes.

The recruitment of GPs from abroad offers the prospect of achieving sufficient supply to meet demand. For this intervention to be fully successful would require the recruitment of an average of 20 GPs per annum which is 0.8% of the current workforce. Using the lower recruitment rate of 10 GPs per annum entails an undersupply from 2012 to 2020. Such new GPs would more easily join existing profitable practices that already have a medical card contract with the government [25]. Joining practices in more deprived areas may be less attractive. Some healthcare systems provide a Government start-up package for new doctors in exchange for service to underserved communities but this is not currently the case in Ireland. Recruitment from within the European Union is preferable for ethical reasons, given the world-wide shortage of doctors. Recruitment from already under-served low and middle income countries runs counter to the focus of the WHO of improving the retention of health professionals in aid-recipient countries [26].

Encouraging GPs to retire later appears to have little impact on supply as it is short-lived and enables supply to meet demand for the next two or three years. This study did not investigate how to incentivise later retirement so it is not clear what resources might be needed to achieve these gains or whether they are even feasible. Given the limited impact of the later retirement it would seem a better prospect to invest limited resources into training new GPs rather than retaining older ones.

The final policy intervention modelled, nurse substitution, is perhaps the most difficult to assess. This study examined the potential for nurses to take up the role of a general practitioner. It is unclear how much of a GP's workload could realistically be provided by nurses and this will be highly dependent on the rules governing prescribing in any given country. Internationally most prescribing nurses are based in hospitals. It is unlikely that they could provide 50% of care normally provided by a GP in the short to medium term. Maynard discusses the importance of substitution of health professionals where there are shortages but such a strategy implies both that there are sufficient numbers of substitutes and that they can be trained quickly, adequately indemnified and supervised more cheaply and safely [27]. Irish forecasts suggest that there will be a shortfall in the supply of domestically trained nurses in the coming decade and that is without a substantial number transferring to a general practice setting [28]. A recent literature review by Normand and Stokes shows that while it may be feasible to handover some tasks, maintaining quality may still require supervision and oversight from GPs [29]. Given that profit maximisation is a key goal as in the Irish market, nurse substitution may be a problematic solution to shortages. GPs are able to demand higher fees, and higher profits, for their *own *services and might therefore be resistant to opening the profession up to less expensive nurses. Another significant issue is referral with evidence from the US suggesting that role substitution has led to increases in rehospitalisation and in use of specialists [30].

Nurse substitution may pose difficulties with regard to integration of two different disciplines. While there are approximately 1050 practice nurses already working in Ireland (approximately 25 per 100,000 persons), the majority are part-time. Under the present system practice nurses are largely funded by practices so small practices are less likely to employ a practice nurse. Almost one third of Irish general practices are single-handed of which 55% do not have access to a practice nurse and may not have the premises to accommodate them. The present study estimated the impact of an additional 50 nurses per year being recruited into general practice which would lead to a 60% increase in the number of nurses in 13 years. These nurses would be providing between 4% and 9% of GP care, depending on the assumptions regarding equivalence. Such a large shift in the make-up of general practice would doubtless entail a very large change in the manner and method of service delivery. A substantial increase in practice nurses might allow GPs to concentrate solely on more complex chronic disease management with other work being given to other members of the primary care team. However, it is clear that pursuing nurse substitution would necessitate a comprehensive review of both the general practice and nursing workforce [31].

The modelling strategies used in this study could equally be applied in other clinical specialties. In fact, taking a cross-specialty approach would be desirable. Depending on the ease with which foreign clinicians can enter a health service, the workforce could be modelled as a closed system. Increasing recruitment in one specialty is likely to reduce recruitment in another, potentially compounding workforce problems rather than solving them. In the present study for example, an intervention proposes to recruit nurses into general practice with clear implications for the nurse workforce. If nurse substitution was to be adopted as policy it would be vital to model the impact on nursing and nurse training to ensure adequate steps are taken to protect service provision overall. Workforce modelling across disciplines provides an opportunity to develop a more unified and cohesive approach to developing health services rather than viewing specialties as isolated units in competition with each other.

The main limitation of this study is the assumption on the degree of equivalence between practice nurses and GPs have a bigger impact on results as it clearly dictates the number of practice nurses needed to fill the gap in supply. There is little literature available to determine a more accurate value for equivalence and a stated strategy of nurse substitution might enable greater role substitution than is in place at present. For the purposes of this study, the scale of practice nurse recruitment required given two equivalence values has been shown. The reliance on assumptions about the demographic profile of practice nurses and GPs recruited from abroad is noted but of less importance. The small difference in delivered sessions between males and females ensure that assumptions about the gender balance of additional recruits should not have a marked impact on the results. The lack of regional data has meant that the potential of each intervention to impact on geographic differences in supply could not be tested. Some interventions may serve to boost supply in urban areas when the greatest shortfalls may actually occur in rural areas and a more formal assessment of interventions should incorporate geographic data. The projected demand for services is based on the assumption that GP visit rates will remain stable by age and sex. With concerns about increasing obesity and the impact this will have on the future prevalence of related illnesses, it is likely that morbidity and the need for general practice services will be higher than at present.

Due to the scale of the predicted shortfall in GP supply, use of a combination of interventions would be more pragmatic than adoption of a single intervention policy. For example, foreign recruitment might not be seen as viable in the long term and nurse substitution might result in a more sustainable solution. However, the transition to nurse substitution is unlikely to happen at the rate required for supply to meet demand so the recruitment of GPs from abroad may provide a rapid response to boost workforce numbers in the short term. Increasing GP training places is desirable and has already been flagged in policy documents but as a sole response falls short of addressing future shortages. The modelling strategy adopted in this study can be applied to incorporate combinations of interventions and hence to determine a useful composite intervention.

## Conclusions

A shortage of GPs will have a rapid and detrimental impact on population health, cost containment and health service utilisation. Selecting the best approach to dealing with the shortage is a complex issue and will involve a number of approaches. Some solutions, such as incentivising later retirement and recruiting from abroad offer the possibility of a quick response and may provide cover while longer term solutions are sought. Late retirement will only have an impact so long as there is a large cohort of older GPs and foreign recruitment faces competition from other countries. Increased training places will not yield a fast result but reflects an investment in the future while nurse substitution presents the opportunity to reshape general practice to meet the demands of the future.

## Abbreviations

GP: general practitioner

## Competing interests

Conor Teljeur, Stephen Thomas and Tom O'Dowd declare that they have no competing interests. Fergus D. O'Kelly is the Director of the Trinity College Dublin/HSE Specialist Training Programme in General Practice.

## Authors' contributions

All authors jointly developed of the concept of the study. TOD and FDOK acquired data on general practitioner demography and work patterns. CT and ST collected demographic and international comparative data. The authors jointly conceived of the policy interventions. CT developed and conducted the simulation modelling. All authors were involved in drafting, revising and approving the final submitted manuscript.

## Authors' information

Conor Teljeur was part-funded by the Health Research Board of Ireland through the HRB Centre for Primary Care Research (PRIMCARE) under Grant HRC/2007/1

## Pre-publication history

The pre-publication history for this paper can be accessed here:

http://www.biomedcentral.com/1472-6963/10/148/prepub
